# OXA-48-like carbapenemases in *Proteus mirabilis* – novel genetic environments and a challenge for detection

**DOI:** 10.1080/22221751.2024.2353310

**Published:** 2024-05-07

**Authors:** Janko Sattler, Janina Noster, Yvonne Stelzer, Martina Spille, Sina Schäfer, Kyriaki Xanthopoulou, Julian Sommer, Jonathan Jantsch, Silke Peter, Stephan Göttig, Sören G. Gatermann, Axel Hamprecht

**Affiliations:** a Institute for Medical Microbiology, Immunology and Hygiene, University Hospital Cologne and Faculty of Medicine, University of Cologne, Cologne, Germany; b Institute for Medical Microbiology and Virology, University of Oldenburg, Oldenburg, Germany; c Institute of Medical Microbiology and Hygiene, University Hospital Tuebingen, Tuebingen, Germany; d Goethe University Frankfurt, University Hospital, Institute of Medical Microbiology and Infection Control, Frankfurt am Main, Germany; e National Reference Laboratory for Multidrug-Resistant Gram-negative Bacteria, Department of Medical Microbiology, Ruhr-University Bochum, Bochum, Germany; f German Centre for Infection Research (DZIF)

**Keywords:** *Proteus mirabilis*, OXA-48, OXA-181, OXA-162, carbapenemase-producing Enterobacterales

## Abstract

OXA-48-like enzymes represent the most frequently detected carbapenemases in Enterobacterales in Western Europe, North Africa and the Middle East. In contrast to other species, the presence of OXA-48-like in *Proteus mirabilis* leads to an unusually susceptible phenotype with low MICs for carbapenems and piperacillin-tazobactam, which is easily missed in the diagnostic laboratory. So far, there is little data available on the genetic environments of the corresponding genes, *bla*_OXA-48_-like, in *P. mirabilis.* In this study susceptibility phenotypes and genomic data of 13 OXA-48-like-producing *P. mirabilis* were investigated (OXA-48, *n* = 9; OXA-181, *n* = 3; OXA-162, *n* = 1). Ten isolates were susceptible to meropenem and ertapenem and three isolates were susceptible to piperacillin-tazobactam. The gene *bla*_OXA-48_ was chromosomally located in 7/9 isolates. Thereof, in three isolates *bla*_OXA-48_ was inserted into a *P. mirabilis* genomic island. Of the three isolates harbouring *bla*_OXA-181_ one was located on an IncX3 plasmid and two were located on a novel MOB_F_ plasmid, pOXA-P12, within the new transposon Tn*7713*. In 5/6 isolates with plasmidic location of *bla*_OXA-48-_like, the plasmids could conjugate to *E. coli* recipients *in vitro*. *Vice versa*, *bla*_OXA-48_-carrying plasmids could conjugate from other Enterobacterales into a *P. mirabilis* recipient. These data show a high diversity of *bla*_OXA-48_-like genetic environments compared to other Enterobacterales, where genetic environments are quite homogenous. Given the difficult-to-detect phenotype of OXA-48-like-producing *P. mirabilis* and the location of *bla*_OXA-48_-like on mobile genetic elements*,* it is likely that OXA-48-like-producing *P. mirabilis* can disseminate, escape most surveillance systems, and contribute to a hidden spread of OXA-48-like.

## Introduction

Carbapenemases are important mediators of multidrug resistance in Enterobacterales. They can render bacteria resistant to carbapenems and most other *β*-lactam antibiotics. The most frequently detected carbapenemase type in Enterobacterales in Western Europe, North Africa, and the Middle East are OXA-48-like carbapenemases [1]. This group encompasses OXA-48 and variants of the latter with minor changes in the amino acid sequence, e.g. OXA-181 and OXA-162. OXA-48-like are most often reported in *Klebsiella pneumoniae*, *Escherichia coli* and the *Enterobacter cloacae* complex, but much less frequently in other Enterobacterales [2]. Especially in *Proteus mirabilis*, OXA-48-like carbapenemases have only been rarely described [3]. Interestingly, some of the reported isolates exhibited low minimum inhibitory concentrations (MICs) for piperacillin-tazobactam (PIT) [4,5], which is a particularity in carbapenemase-producing Enterobacterales (CPE). OXA-48-like enzymes have only weak hydrolytic activity against carbapenems *in vitro*, which results in low MIC values if no other resistance mechanism is present. The combination of low MIC values for carbapenems and PIT strongly impedes the detection of OXA-48-like-producing *P. mirabilis* in the diagnostic laboratory, since these parameters are often included in diagnostic algorithms for the detection of carbapenemases [6,7]. Nevertheless, the detection of OXA-48-like is crucial, as it has been shown that carbapenems are not an effective treatment option for OXA-48-producing Enterobacterales even if they have MICs within the susceptible range [8]. As *P. mirabilis* is intrinsically resistant to colistin and tigecycline, the choice of last-resort antibiotics is limited in infections with these isolates.

Compared to other carbapenemase genes, the genomic location of *bla*_OXA-48_, the gene coding for OXA-48, is quite homogenous in Enterobacterales. The most common genomic location is on ∼60-70 kb sized IncL plasmids [9] which represent variants of the originally described pOXA-48 [10]. Within these plasmids, *bla*_OXA-48_ is located on variants of the transposon Tn*1999* of which eight have been described until now [11]. However, from the little data published, the genetic environment of *bla*_OXA-48_ in *P. mirabilis* was either located on an unusually large IncL/M plasmid [12] or integrated into the host chromosome [4]. This raises doubts if genomic findings on *bla*_OXA-48_ in other Enterobacterales can be applied to OXA-48-producing *P. mirabilis*.

It is, therefore, unclear whether the low PIT MICs observed in some OXA-48-like-producing *P. mirabilis* isolates are associated with a particular genomic configuration of *bla*_OXA-48_-like-associated environments. Additionally, it is unknown if *bla*_OXA-48_-like are usually located on mobile genetic elements in *P. mirabilis*, and could serve as a hidden source for the spread of *bla*_OXA-48_-like to other Enterobacterales, as postulated very recently [4,5]. In this study, we aim to analyse the genetic environments and transferability of *bla*_OXA-48_-like genes in clinical *P. mirabilis* isolates, together with their susceptibility phenotypes.

## Methods

### Isolates and antibiotic susceptibility testing

Thirteen OXA-48-like-producing *P. mirabilis* isolates from clinical samples were included in the study. They were collected between 2015 and 2021 at the University Hospital Cologne, the University Hospital Frankfurt am Main and the German National Reference Centre for multidrug-resistant Gram-negative bacteria Bochum, including twelve isolates from Germany and one isolate from Austria. The presence of OXA-48-like was confirmed by lateral flow test (RESIST-4 O.K.N.V., Coris BioConcept, Gembloux, Belgium) [13] and PCR (GeneXpert Carba-R assay, Cepheid, Frankfurt, Germany) [14]. Minimal inhibitory concentrations for ertapenem, meropenem, and PIT were determined using broth microdilution (BMD; Micronaut-S, Merlin, Bornheim, Germany). When MICs were outside of the dilution steps of BMD, gradient tests (MIC strips, Liofilchem, Roseto degli Abruzzi, Italy) were used to determine the values. Samples were tested in triplicates and the median MIC value per isolate and antibiotic was reported. The MICs were interpreted for susceptibility according to EUCAST clinical breakpoints version 13.1. To compare MIC values between two isolate groups, the Mann–Whitney test was used and a *p*-value ≤ 0.05 considered statistically significant.

### In vitro plasmid conjugation

Horizontal gene transfer via conjugation was conducted by liquid mating assay as described before [11]. From *P. mirabilis* donors with *bla*_OXA-48_ located on plasmids, conjugation was evaluated employing the sodium azide-resistant acceptor strain *E. coli* J53 and *K. pneumoniae* PRZ. *Vice versa*, conjugation of isogenic *bla*_OXA-48_-carrying plasmids from clinical non-*Proteus* Enterobacterales isolates was performed in parallel into acceptor strains J53 and a sodium azide-resistant *P. mirabilis* strain, further referred to as ARP. Successful conjugation was confirmed by antibiotic susceptibility testing, MALDI-TOF, and whole genome sequencing (WGS). Quantitative conjugation was conducted in three independent runs and the mean conjugation rate and standard deviation was reported.

### Genetic analysis

For genetic analysis, DNA was extracted from pure bacterial cultures using the DNeasy UltraClean Microbial Kit (Qiagen, Hilden, Germany). Whole genome sequencing was performed with short- and long-read sequencing technology. Briefly, libraries were prepared with the native barcoding kits SQK-LSK109 or SQK-NBD114 (Oxford Nanopore Technologies, Oxford, UK), and Illumina DNA Prep (Illumina, San Diego, CA, United States) and sequencing was performed on a Oxford Nanopore PromethION with flow cell type R9.4.1 or R10.4.1 and on a Illumina NextSeq500. Genomes were assembled from long-reads with Trycycler [15] followed by long-read polishing with medaka [16] and short-read polishing with polypolish [17]. The classes and abundances of resistance genes and plasmids were determined using the ResFinder and PlasmidFinder databases [18,19]. Plasmids that could not be classified by PlasmidFinder were further characterized using MOBscan [20] and MOB-suite [21]. Sequences were annotated using the automated PGAP pipeline of NCBI [22], as well as manually. For the phylogenetic analysis all publicly available sequences of OXA-48-like-producing *P. mirabilis* from GenBank were included. A cgMLST scheme was created with chewBBACA [23]. Allele calling was performed with a threshold of 95% loci presence in the test dataset, resulting in 2,334 loci. A minimum spanning tree was created from the allelic profiles using GrapeTree [24]. For isolates of the same ST type, whole genome SNP analysis was performed with CSIPhylogeny 1.4 [25]. The GenBank accession numbers of external isolates included in the phylogenetic analysis are shown in Supplementary Table 1. Comparative genome graphs were created with BRIG 0.95 [26] and clinker [27].

### Data  availability

The complete nucleotide sequence assemblies of all OXA-48-like-producing *P. mirabilis* were deposited at DDBJ/ENA/GenBank under BioProject no. PRJNA982542. Accession numbers are listed in Supplementary Table 2.

#### Ethics

Due to the retrospective design of this study which involved only bacterial isolates, no approval by an ethics committee is required according to § 15 of the professional code for physicians of North Rhine-Westphalia. This was confirmed by the ethics committee of the University Hospital Cologne under the statement number “23-1212-retro”.

## Results

### Isolates and antibiotic susceptibility testing

Of the 13 isolates included in this study, nine produced OXA-48, three OXA-181, and one OXA-162 (Table 1). For most isolates, ertapenem and meropenem MICs were between 0.25 and 1 mg/L; 10/13 isolates (77%) were susceptible to ertapenem, and 11/13 (85%) to meropenem. Exceptionally, P1, P12, and P13 showed elevated MICs for meropenem and/or ertapenem. MICs for PIT were >64 mg/L in 8/13 isolates (62%), while MICs in the susceptible range (≤ 8 mg/L) were recorded in three isolates (P1, P4, and P5).

**Table 1.** Genotypic and phenotypic characteristics of OXA-48-like-producing *P. mirabilis* isolates from this study.

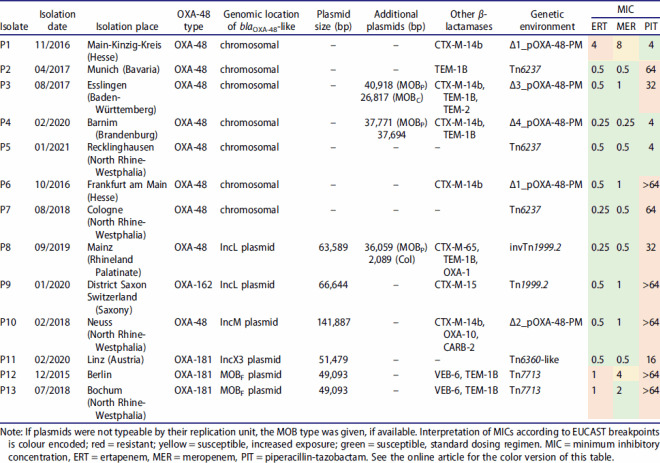

### Phylogeny

**Figure 1.**
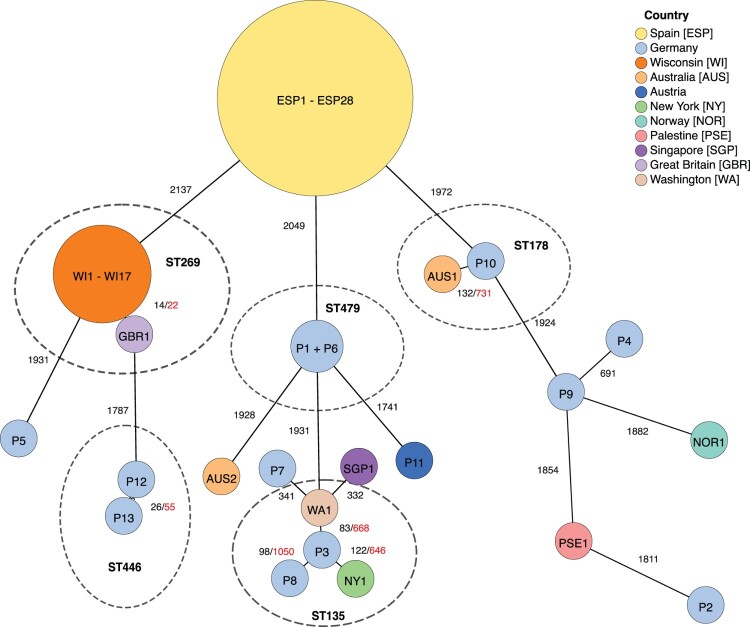
**Maximum-likelihood cgMLST-based phylogeny of OXA-48-like-producing *P. mirabilis* from this study and external sequences from GenBank**. The distance of the tree branches represents allelic differences in the cgMLST scheme (in total 2,334 loci). Nodes with close epidemiological relationship were combined in one node (cut-off: 12 allelic differences). Isolates with the same ST type are indicated by dotted circles. Within the same ST type, genomic distances are also expressed as SNPs from whole genome phylogeny (red numbers).

A minimum spanning tree was created for allelic differences in the cgMLST, including all isolates from this study (*n* = 13) and non-redundant publicly available sequences on GenBank of OXA-48-like-producing *P. mirabilis* (*n* = 43) (Figure 1). While most isolates were only distantly related, some isolates formed small clusters within the same ST type. Amongst these, P1 and P6 (ST479) showed no allelic difference and only 11 SNPs difference in the whole genome and were isolated from adjacent geographical regions (Rhine-Main area), about one month apart, making an epidemiological relationship likely. The other clusters were P12 and P13 (ST446, 26 allelic and 55 SNP difference), P3, P8 and the external isolates NY1 and WA1 (ST135, 83–122 allelic and 646–1050 SNP differences) and P10 and AUS1 (ST178, 132 allelic and 731 SNP differences). They showed no close regional and temporal context.

### Genetic support of bla_OXA-48_-like

In all isolates, the genetic environment of *bla*_OXA-48_-like was examined using hybrid assemblies. In 7/9 OXA-48-producing isolates, *bla*_OXA-48_ was located on the chromosome. In the other two isolates, *bla*_OXA-48_ was located on an IncL or IncM plasmid, respectively. The closely related gene *bla*_OXA-162_ was also situated on an IncL plasmid in one isolate. The gene *bla*_OXA-181_ was located on an IncX3 plasmid in one isolate and a novel MOB_F_ plasmid in two isolates (Table 1).

### Chromosomal location of bla_OXA-48_

**Figure 2.**
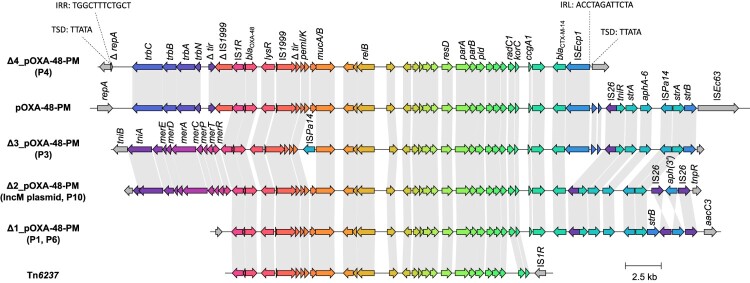
**Variants of genetic environments of *bla*_OXA-48_ in *P. mirabilis* compared to the plasmid pOXA-48-PM***.* Grey arrows indicate mobile genetic elements or associated genes. Unlabelled arrows indicate genes coding for hypothetical proteins. IRL/IRR = left and right inverted repeats. TSD = target site duplications.

In the seven isolates with chromosomal localization, different genetic environments of *bla*_OXA-48_ were observed. In isolates P2, P5, and P7, *bla*_OXA-48_ was integrated via the IS*1R*-bounded composite transposon Tn*6237* (Figure 2). For the other four isolates, the genetic environments of *bla*_OXA-48_ show high sequence homology with different genetic subsections of the plasmid pOXA-48-PM, which was the first *bla*_OXA-48_-carrying plasmid described in *P. mirabilis* [12] (Figure 2). These four genomic regions are therefore referred to as Δ1_pOXA-48-PM to Δ4_pOXA-48-PM.

In isolate P3, Δ3_pOXA-48-PM spans from IS*1R* next to *bla*_OXA-48_ until *strB*, but harbours an additional IS*Pa14*, truncating *mucA*. Isolate P1 and P6 harbour Δ1_pOXA-48-PM, which is a variant of Δ3_pOXA-48-PM missing the additional IS*Pa14* as well as IS*Ecp1* upstream of *bla*_CTX-M-14_ together with two ORFs. Isolate P4 hosts Δ4_pOXA-48-PM, which has its starting point further upstream of the *bla*_OXA-48_-harbouring invTn*1999.2*, spanning the whole pOXA-48-PM region from *ΔrepA* until IS*Ecp1*.

**Figure 3.**
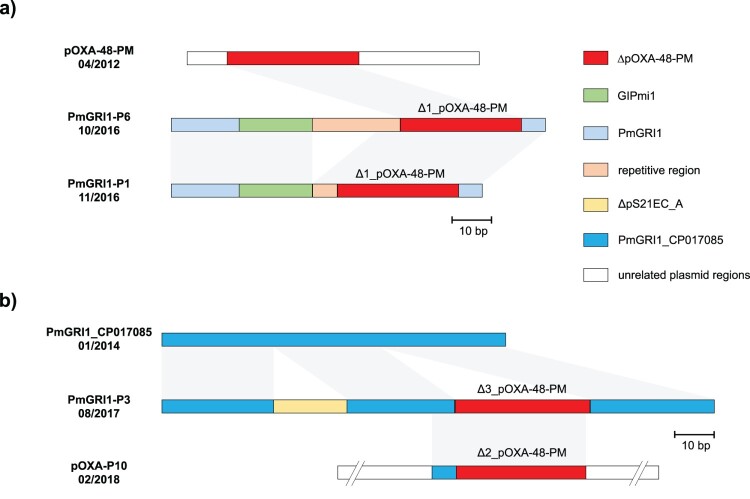
**Graphical representation of the novel genomic islands PmGRI1-P1/P6 (a) and PmGRI1-P3 and the plasmid pOXA-P10 (b).** Genetic structures are colour encoded according to the legend. Grey areas between the structures indicate regions of high sequence homology. Numbers below the name label describe the isolation date of the according isolate.

In isolates harbouring Tn*6237* and Δ4_pOXA-48-PM, all genetic environments of *bla*_OXA-48_ were chromosomally integrated into different sites of the conserved genetic region of *P. mirabilis*. For Δ4_pOXA-48-PM the integrated sequence is bounded by 12 bp imperfect inverted repeats containing five mismatches and flanked by 5 bp target site duplications, indicating one-ended transposition by IS*Ecp1* (Figure 2). Tn*6237* was integrated in different genetic sites in the three isolates, expectedly resulting in different 9 bp sized target site duplications. In contrast, in the other three isolates with chromosomal ΔpOXA-48-PM variants (P1, P3, and P6), the genetic environment of *bla*_OXA-48_ was integrated in novel variants of the genomic island PmGRI1 [28], not flanked by target site duplications. The GC content of the novel genomic islands was 53.2-54.9% compared to 38.9% for the *P. mirabilis* reference strain HI4320. Like PmGRI1, these genomic islands are integrated at the 3′ end of tRNA-Sec in the vicinity of the *prlC* gene and share the same backbone, including complete homology within the integrase gene. Therefore, they are referred to as PmGRI1-P1, -P3, and -P6. PmGRI1-P6 is 96,474 bp in size. Besides the genomic island backbone of PmGRI1 and ΔpOXA-48-PM, it contains sequence regions homologous with parts of the genomic island GIPmi1 (accession number MF490433) (Figure 3). PmGRI1-P1 has a highly similar composition to PmGRI-P1, but an excision in a repetitive region resulted in a smaller size of 77,906 bp. PmGRI1-P3 shows high sequence homology with a PmGRI1 variant published under the accession number CP017085 [29]. It differs from the latter by the addition of ΔpOXA-48-PM as well as parts of an *E. coli* plasmid, pS21EC_A (accession number CP076690) [30], resulting in a size of 139,172 bp.

### Plasmidic location of bla_OXA-48/162_

**Figure 4.**
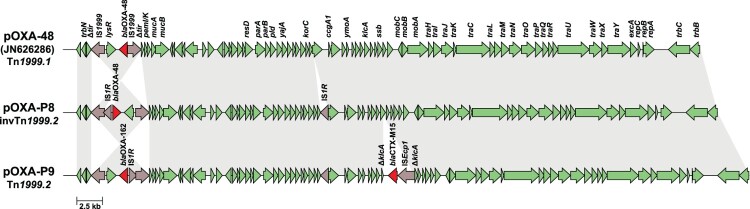
**Plasmid variants of pOXA-48 found in two *P. mirabilis* isolates.** Grey arrows = mobile genetic elements, red arrows = antibiotic resistance genes, green arrows = other genes or open reading frames. Unlabelled arrows indicate genes coding for hypothetical proteins.

In P8 and P9, *bla*_OXA-48_-like were located on IncL plasmids, further referred to as pOXA-P8 and pOXA-P9. In pOXA-P8, *bla*_OXA-48_ was located on an inverted Tn*1999.2*, and, compared to the originally described pOXA-48, the plasmid contains an additional IS*1R* element downstream of *korC* (Figure 4). In pOXA-P9, *bla*_OXA-162_ was located on a Tn*1999.2* transposon. Compared to pOXA-P8, this plasmid harbours an additional 3,049 bp IS*Ecp1* transposition unit carrying *bla*_CTX-M-15_ as cargo gene, truncating *klcA*. Both IncL plasmids were transferable from their *P. mirabilis* donor to *E. coli* J53 via conjugation and pOXA-P9 was also transferable to *K. pneumoniae* PRZ. The IncM plasmid pOXA-P10 did not conjugate to J53 or PRZ in our *in vitro* assay.

### Plasmidic location of bla_OXA-181_

**Figure 5.**
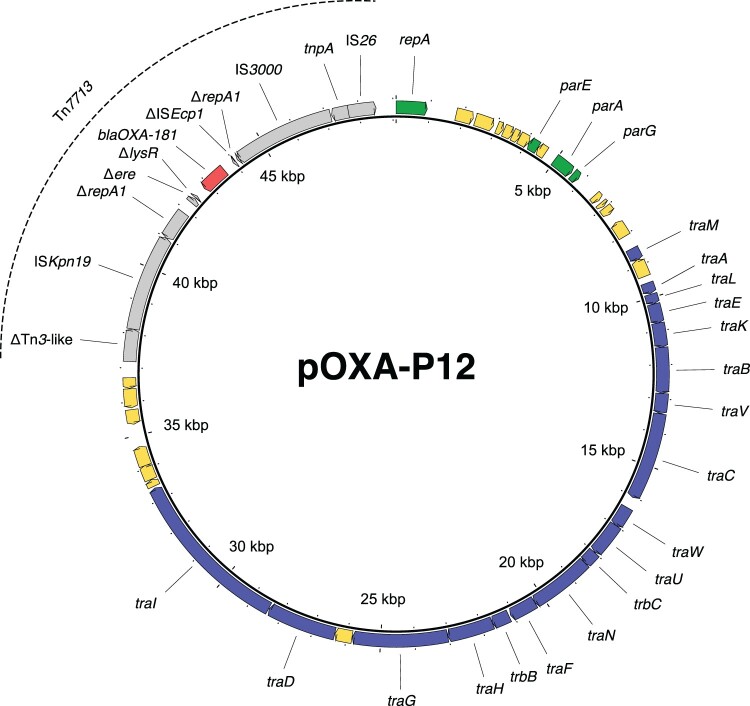
**The novel MOB_F_ plasmid pOXA-P12, harbouring *bla*_OXA-181_ within the novel transposon Tn*7713*.** Red arrow = *bla*_OXA-48_, grey arrows = other parts of Tn*7713*, green arrows = genes associated with plasmid replication, blue arrows = genes associated with plasmid transfer, yellow arrows = genes coding for hypothetical proteins.

Three isolates harboured *bla*_OXA-181_. In isolate P11, *bla*_OXA-181_ was located on a 51,479 bp-sized IncX3 plasmid, further referred to as pOXA-P11. This IncX3 plasmid is identical to plasmids described in other species, e.g. pOXA-181_14828 (accession number KP400525) [31]. In the other two isolates, P12 and P13, *bla*_OXA-181_ is located on a novel 49,093 bp-sized plasmid, further referred to as pOXA-P12 (Figure 5). This plasmid could not be typed to a known Inc group, but the relaxase gene could be assigned to the MOB_F_ type. The plasmids of these two isolates show 100% sequence identity, despite being isolated three years apart.

**Figure 6.**
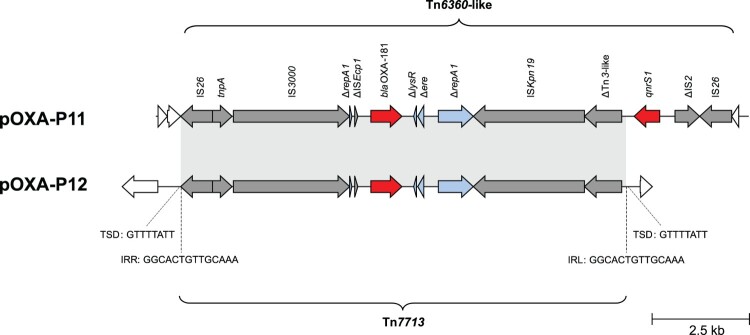
**Tn*6360*-like variants in *bla*_OXA-181_-harbouring *P. mirabilis***. Grey arrows = mobile genetic elements or associated genes, red arrows = antibiotic resistance genes, blue arrows = other genes or gene fragments, white arrows = genes outside of the transposon structure. IRL/IRR = left and right inverted repeats, TSD = target site duplications.

In all three isolates, *bla*_OXA-181_ was surrounded by a genetic environment related to the Tn*6360* transposon (Figure 6). In pOXA-P11, the genetic environment is identical to that of a Tn*6360* variant described before [31] and is here referred to as Tn*6360*-like. In pOXA-P12, *bla*_OXA-181_ was found on a novel variant of Tn*6360*-like. This variant is truncated upstream of the ΔTn*3*-like sequence and was assigned the name Tn*7713* by the transposon registry [32] (Figure 6). Tn*7713* is bounded by 14 bp inverted repeats and flanked by 8 bp target site duplications.

Both *bla*_OXA-181_-carrying plasmid types could conjugate to *E. coli* J53, while only pOXA-P11 conjugated to PRZ. WGS analysis of the transconjugants showed that pOXA-P11 and pOXA-12 stayed intact during the transfer except for one conjugation experiment, where plasmid pOXA-P12 was integrated into the chromosome of the recipient J53, bounded by two copies of IS*26* and flanked by 8 bp target site duplications.

### Plasmid conjugation into P. mirabilis

**Figure 7.**
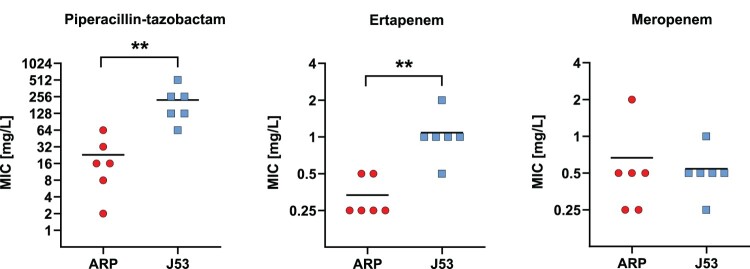
**Minimal inhibitory concentrations (MICs) of piperacillin-tazobactam, ertapenem and meropenem for OXA-48-producing transconjugants.** Transconjugants comprise *P. mirabilis* ARP (red dots, *n* = 6) and *E. coli* J53 (blue squares, *n* = 6), sharing isogenic pOXA-48-like plasmid variants. Black bars indicate the median. Two asterisks indicate a statistically significant difference of *p* ≤ 0.01.

Variants of pOXA-48 were conjugated from seven Enterobacterales isolates (*K. pneumoniae, E. coli,* and *Citrobacter freundii*) in parallel to the recipients *P. mirabilis* ARP and *E. coli* J53. Some of the donor isolates have been characterized previously (KP17051, EC8448 [11], EC7215 [33]). In 6/7 isolates, transconjugants of both recipients harboured intact pOXA-48-like plasmids and no other plasmids. In one ARP transconjugant, Tc-KP17051-ARP, genomic analysis revealed a truncated *bla*_OXA-48_-carrying plasmid of 18 kb size in the initial conjugation experiment, but the plasmid remained intact during transfer in follow-up experiments. Hence, this isolate pair was excluded from the comparative MIC analysis. For the other six transconjugant pairs, MICs for PIT and ertapenem were significantly lower in ARP transconjugants than in J53 transconjugants, while they were on a comparable level for meropenem (Figure 7, Supplementary Table 3). The pOXA-48 conjugation rates to J53 and ARP were compared from two representative donors, *E. coli* EC7215 and *K. pneumoniae* KP12369. They were significantly higher when transferred into J53 (3.59 × 10^−4^ [SD 1.37 × 10^−4^] and 1.7 × 10^−3^ [SD 2 × 10^−3^]) than into ARP (1.39 × 10^−6^ [SD 1.39 × 10^−6^] and 4.94 × 10^−7^ [SD 1.86 × 10^−7^]).

## Discussion

In this study, we characterized *bla*_OXA-48_-like-producing *P. mirabilis* and reported on so far undescribed plasmids and transposons.

### High ratio of chromosomal bla_OXA-48_ integration

With 7/9 OXA-48-producing isolates, this study demonstrated a high rate of chromosomally integrated *bla*_OXA-48_ in *P. mirabilis* compared to other Enterobacterales [33]. Only in one isolate, *bla*_OXA-48_ had the typical genomic location within a Tn*1999*-like transposon on an IncL plasmid, which is usually present in *K. pneumoniae* or *E. coli*. From the scarce available data on *bla*_OXA-48_ in *P. mirabilis*, two studies found *bla*_OXA-48_ located on IncL/M plasmids [12,34], while a third study detected *bla*_OXA-48_ on the chromosome of a *P. mirabilis* outbreak clone in Spain [4].

Our results might indicate, that the genetic environment of *P. mirabilis* is highly supportive for chromosomal integration of *bla*_OXA-48_.

First, based on our data and the studies mentioned above, invTn*1999.2* seems to be the exclusive Tn*1999* variant in *bla*_OXA-48_-carrying *P. mirabilis* isolates. This is in contrast to other Enterobacterales, where Tn*1999.2* and Tn*1999.1* prevail [33]. This can explain the high ratio of chromosomal insertion via Tn*6237* in *P. mirabilis,* where invTn*1999.2* leads to a configuration that allows for pseudo-compound transposition of the *bla*_OXA-48_ environment via two copies of IS*1R*. While this is only occasionally described in other Enterobacterales, mainly in *E. coli* ST38 [35], this was observed at a high ratio (*n* = 3) in this study. As the respective isolates are phylogenetically distant and the insertions sites of Tn*6237* differs between isolates, this indicates several integration events instead of clonal spread.

Second, *P. mirabilis* strains contain genomic islands at high frequency, e.g. of the type SGI1[36], PGI1 [37], PGI2 [38], GIPmi1 [39] or PmGRI1 [28]. These genomic islands, including the novel PmGRI1 variants described in this study, are rich in mobile genetic elements, such as transposons and insertion sequences, making them a potential hot spot for the integration of foreign genome sequences. The integration of DNA segments containing resistance genes in the chromosome via homologous recombination has been reported, e.g. for *Acinetobacter baumannii* [40] and subsequent integration events can lead to Russian doll-like structured genomic islands in this species [41]. Our study suggests a similar genomic island evolution for *P. mirabilis*, as illustrated in Figure 3, where some PmGRI1 variants might have evolved from each other and other genomic structures. Furthermore, novel variants of PmGRI1 with different genetic content have been described recently, indicating an ongoing evolution. Isolates harbouring these PmGRI1 variants were obtained from human samples [42,43], swine [44], and chicken [28]. This emphasizes the important link of *P. mirabilis* for the spread of multidrug resistance between animals and humans, as highlighted in previous studies [45].

Another possible explanation for the high rate of chromosomal integration of *bla*_OXA-48_ in *P. mirabilis* would be that the *bla*_OXA-48_-carrying plasmids are unstable in this species, therefore selecting for isolates with chromosomal location. This hypothesis has not been tested in this study, which is a limitation.

### Genomic environments of bla_OXA-181_ in P. mirabilis

The presence of a *bla*_OXA-181_-carrying IncX3 plasmid in *P. mirabilis* identical to one described in *E. coli* before [31], as well as a large number of plasmid sequence matches in other Enterobacterales species on GenBank, indicates interspecies spread which could also be shown *in vitro* in this study. Possibly *bla*_OXA-181_ was transferred from the IncX3 plasmid to the MOB_F_ plasmid, pOXA-P12, via Tn*7713*. This plasmid was also capable of interspecies conjugation *in vitro*. The plasmid pOXA-P12 was detected in two isolates, which were isolated with a time difference of 2.5 years and had no spatial relationship. This could indicate either repeated integration events or high stability of this plasmid in *P. mirabilis*. Interestingly, the initially described *bla*_OXA-181_-carrying ColE2 plasmid (synonym: ColKP3) was not self-conjugative, at least *in vitro* [46]. However, after mobilization of the genetic environment onto the IncX3 plasmid it could conjugate to *E. coli* J53 and *K. pneumoniae* PRZ *in vitro* in the absence of other plasmids. Mobilization of *bla*_OXA-181_ onto self-transmissible plasmids could be an alarming event, as this could support the hypothesis of a silent spread of *bla*_OXA-48_-like through *P. mirabilis*.

### Low MICs for carbapenems and piperacillin-tazobactam

Carbapenem and PIT MICs were unevenly distributed among the clinical isolates. As 10/13 isolates exhibit only slightly elevated carbapenem MICs (0.25–1 mg/L), the presence of OXA-48-like might be missed in the diagnostic laboratory, depending on the screening algorithm used.

MICs for PIT are usually strongly elevated in OXA-48-like-producing Enterobacterales, typically >128 mg/L [47]. In this study, three isolates exhibited PIT MICs in the susceptible range, all of which harboured *bla*_OXA-48_ on their chromosome. This finding could indicate an association between the chromosomal location of *bla*_OXA-48_ and low PIT MICs. Low PIT MIC could be mediated by lower copy numbers of chromosomal *bla*_OXA-48_ compared to plasmidic location, which is supported by the relative contig depth estimation of Unicycler in 5/6 *bla*_OXA-48_-like-harbouring plasmids (Supplementary Table 4). However, susceptibility to PIT could not be solely inferred from chromosomal location even in the absence of other *β*-lactamase genes (e.g. P5 and P7). Furthermore, conjugation experiments showed that the presence of a pOXA-48 variant in a *P. mirabilis* recipient also led to PIT MICs in the susceptible range in some isolates, indicating that PIT susceptibility is not strictly associated with chromosomal location. A mutation in the *bla*_OXA-48_ promoter region can be a reason for PIT susceptibility in OXA-48-producing *P. mirabilis* [4]; however, this was ruled out in our isolates. Overall, these data indicate that in *P. mirabilis* the susceptibility phenotype for carbapenems and PIT might be influenced by non-enzymatic resistance mechanisms, of which, for example, alterations of the penicillin-binding proteins as well as outer membrane proteins have been described [48,49], which should be analysed in future studies.

### Interspecies conjugation of bla_OXA-48_-like-carrying plasmids

While interspecies conjugation of the highly conjugative pOXA-48-like plasmids in other *bla*_OXA-48_-carrying Enterobacterales has been described as a main mediator for the spread of OXA-48 [33,50], this has not been comprehensively studied in *P. mirabilis*.

This study showed interspecies conjugation of *bla*_OXA-48_-carrying plasmids in both directions; from *P. mirabilis* into *E. coli* and from different Enterobacterales species into *P. mirabilis*. Conjugation of other carbapenemase-carrying plasmids from *E. coli* to *P. mirabilis* (reference strain CIP103181) has been described before at low transfer frequencies compared to other Enterobacterales recipients [51]. However, this is to the best of our knowledge the first description of conjugation of the widespread pOXA-48-like plasmids to *P. mirabilis.* These data indicate that *P. mirabilis* participates in the spread of *bla*_OXA-48_-like-carrying plasmids between Enterobacterales *in vivo*. In combination with a difficult-to-detect resistance phenotype, this could be an alarming finding, as *bla*_OXA-48_-like-carrying mobile genetic elements could be distributed within the Enterobacterales communities below the radar of surveillance. However, to estimate the burden of *P. mirabilis* in the spread of OXA-48-like, reliable detection methods need to be implemented first in diagnostic laboratories. An algorithm with 100% sensitivity in the detection of carbapenemase-producing *P. mirabilis* has been described recently and implementation might be advocated in the face of the results of this study [5].

### Limitations

A limitation of this study is that it only comprises isolates from Germany and Austria. The genetic environment of *bla*_OXA-48_-like in *P. mirabilis* seems to be diverse, and the genetic background of the included isolates differs from the isolates published so far from other countries. Furthermore, other OXA-48-like subtypes have been described in *P. mirabilis* from other countries, such as OXA-204 in France [52] and OXA-244 in Russia [34]. Therefore, it is likely that in other geographic regions, different genetic environments of *bla*_OXA-48_-like are present, which should be further studied.

Another limitation is that the species-specific factors contributing to different MICs in different species with an isogenic plasmid that were observed in this study have not been further investigated.

## Conclusion

This study showed that the genetic environments of *bla*_OXA-48_-like in *P. mirabilis* are essentially different from those seen in other Enterobacterales, but *bla*_OXA-48_-like can be transferred between *P. mirabilis* and other Enterobacterales by various mobile genetic elements. Together with a hard-to-detect phenotype, *P. mirabilis* therefore likely contributes to the global spread of OXA-48-like enzymes in Enterobacterales.

## Supplementary Material

Supplemental Material
