# Supplementary material for: OXA-48-like carbapenemases in Proteus mirabilis – novel genetic environments and a challenge for detection
Source: Emerg Microbes Infect. 2024 May 7;13(1):2353310. doi: 10.1080/22221751.2024.2353310 (PMC11123474; doi:10.1080/22221751.2024.2353310)
Supplement: Supplemental Material [file TEMI_A_2353310_SM5273.docx]

OXA-48-like carbapenemases in *Proteus mirabilis* - novel genetic
environments and a challenge for detection

Janko Sattler^1,2,6*^, Janina Noster^2^, Yvonne Stelzer^2^, Martina Spille^2^, Sina Schäfer^1,6^, Kyriaki Xanthopoulou^1,6^, Julian Sommer^4^, Jonathan Jantsch^1,6^, Silke Peter^3,6^, Stephan Göttig^4^, Sören G. Gatermann^5^, Axel Hamprecht^1,2,6^

**^Supplementary material^**

**Supplementary Table 1:** GenBank accession numbers and country of origin of external sequences included in the phylogenetic analysis.

| **ID used in figure** | **GenBank accession number** | **Country of origin** |
| --- | --- | --- |
| AUS1 | GCA_016065595 | Australia |
| AUS2 | GCA_025974925 | Australia |
| GBR1 | GCA_022110455 | Great Britain |
| ESP1 | GCA_023242175 | Spain |
| ESP2 | GCA_023242195 | Spain |
| ESP3 | GCA_026954605 | Spain |
| ESP4 | GCA_026955875 | Spain |
| ESP5 | GCA_026955925 | Spain |
| ESP6 | GCA_026955965 | Spain |
| ESP7 | GCA_026956055 | Spain |
| ESP8 | GCA_026956115 | Spain |
| ESP9 | GCA_026957015 | Spain |
| ESP10 | GCA_026957035 | Spain |
| ESP11 | GCA_026957055 | Spain |
| ESP12 | GCA_026957065 | Spain |
| ESP13 | GCA_026957095 | Spain |
| ESP14 | GCA_026957175 | Spain |
| ESP15 | GCA_026957555 | Spain |
| ESP16 | GCA_026957575 | Spain |
| ESP17 | GCA_026957655 | Spain |
| ESP18 | GCA_026957675 | Spain |
| ESP19 | GCA_026957695 | Spain |
| ESP20 | GCA_026958975 | Spain |
| ESP21 | GCA_026960155 | Spain |
| ESP22 | GCA_026960195 | Spain |
| ESP23 | GCA_026960215 | Spain |
| ESP24 | GCA_026960235 | Spain |
| ESP25 | GCA_026960255 | Spain |
| ESP26 | GCA_026960275 | Spain |
| ESP27 | GCA_026961225 | Spain |
| ESP28 | GCA_026962515 | Spain |
| NOR1 | GCA_025880885 | Norway |
| NY1 | GCA_032704585 | New York |
| PSE1 | GCA_000770765 | Palestine |
| SGP1 | GCA_023035995 | Singapore |
| WA1 | GCA_020777215 | Washington |
| WI1 | GCA_031107755 | Wisconsin |
| WI2 | GCA_032262725 | Wisconsin |
| WI3 | GCA_032262765 | Wisconsin |
| WI4 | GCA_033011795 | Wisconsin |
| WI5 | GCA_033011895 | Wisconsin |
| WI6 | GCA_033182205 | Wisconsin |
| WI7 | GCA_033182245 | Wisconsin |

**Supplementary Table 2:** GenBank accession numbers of the isolates’ sequence assemblies. TBD = to be determined (accession number not available yet at the time point of resubmission of the manuscript).

| **Isolate** | **Accession number** |
| --- | --- |
| **P1** | CP154802 |
| **P2** | CP148143 |
| **P3** | CP151676-CP151678 |
| **P4** | JAWIPV000000000 |
| **P5** | CP148142 |
| **P6** | CP154801 |
| **P7** | CP154800 |
| **P8** | CP151672-CP151675 |
| **P9** | CP148140-CP148141 |
| **P10** | CP148138-CP148139 |
| **P11** | JASZXX000000000 |
| **P12** | CP148134-CP148135 |
| **P13** | CP148136-CP148137 |
| **Tc-CF5727-J53** | JBBJDC000000000 |
| **Tc-CF5727-ARP** | JBBJDB000000000 |
| **Tc-EC8448-J53** | JBBJDA000000000 |
| **Tc-EC8448-ARP** | JBBJCZ000000000 |
| **Tc-KP12369-J53** | JBBJCY000000000 |
| **Tc-KP12369-ARP** | JBBJCX000000000 |
| **Tc-KP18278-J53** | JBBJCW000000000 |
| **Tc-KP18278-ARP** | JBBJCV000000000 |
| **Tc-KP3368-J53** | JBBJCU000000000 |
| **Tc-KP3368-ARP** | JBBJCT000000000 |
| **Tc-EC7215-J53** | JBBJCS000000000 |
| **Tc-EC7215-ARP** | JBBJCR000000000 |
| **Tc-KP17051-J53** | JBCPSE000000000 |
| **Tc-KP17051-ARP** | JBCPSD000000000 |
| **Tc-P8-J53** | JBBJCQ000000000 |
| **Tc-P9-J53** | JBBJCP000000000 |
| **Tc-P11-J53** | JBBJCO000000000 |
| **Tc-P12-J53** | JBCPSC000000000 |
| **Tc-P9-PRZ** | JBCPSB000000000 |
| **Tc-P11-PRZ** | JBCPSA000000000 |

**Supplementary Table 3:** MIC values of transconjugants in *P. mirabilis* ARP (n=6) and *E. coli* J53 (n=6), sharing isogenic pOXA-48 plasmid variants.

| **Isolate** | **Piperacillin-tazobactam** | **Ertapenem** | **Meropenem** |
| --- | --- | --- | --- |
| **Tc_KP12369_J53** | 64 | 1 | 0.5 |
| **Tc_KP12369_ARP** | 2 | 0.25 | 0.25 |
| **Tc_CF5727_J53** | 256 | 1 | 0.5 |
| **Tc_CF5727_ARP** | 32 | 0.25 | 0.5 |
| **Tc_KP18278_J53** | 128 | 0.5 | 0.5 |
| **Tc_KP18278_ARP** | 16 | 0.5 | 0.25 |
| **Tc_KP3368_J53** | 256 | 1 | 0.5 |
| **Tc_KP3368_ARP** | 64 | 0.25 | 0.5 |
| **Tc_EC8448_J53** | 128 | 1 | 0.25 |
| **Tc_EC8448_ARP** | 8 | 0.5 | 2 |
| **Tc_EC7215_J53** | 512 | 2 | 1 |
| **Tc_EC7215_ARP** | 16 | 0.25 | 0.5 |

**Supplementary Table 4:** Relative contig depth estimation of the *bla*_OXA-48_-like-harbouring plasmids by Unicycler.

| **Isolate** | **Plasmid type** | **Relative contig depth** |
| --- | --- | --- |
| **P8** | IncL | 1.39x |
| **P9** | IncL | 1.65x |
| **P10** | IncM | 3.50x |
| **P11** | IncX3 | 0.56x |
| **P12** | untypeable | 1.95x |
| **P13** | untypeable | 2.08x |
|  |  |  |
